# Exogenous melatonin delays oxidative browning in litchi during cold storage by regulating biochemical attributes and gene expression

**DOI:** 10.3389/fpls.2024.1402607

**Published:** 2024-06-06

**Authors:** Kilchira A. Marak, Hidayatullah Mir, Mohammed Wasim Siddiqui, Preeti Singh, Fozia Homa, Saud Alamri

**Affiliations:** ^1^ Department of Horticulture (Fruit and Fruit Technology), Bihar Agricultural University, Bhagalpur, Bihar, India; ^2^ Department of Horticulture (Fruit Science), Regional Horticulture Research Sub-Station, Bhaderwah, Sher-e-Kashmir University of Agricultural Sciences and Technology (SKUAST), Jammu, Jammu and Kashmir, India; ^3^ Department of Food Science and Postharvest Technology, Bihar Agricultural University, Bhagalpur, Bihar, India; ^4^ Department of Statistics, Bihar Agricultural University, Bhagalpur, Bihar, India; ^5^ Department of Botany and Microbiology, College of Science, King Saud University, Riyadh, Saudi Arabia

**Keywords:** *Litchi chinensis*, melatonin, pericarp browning, cold storage, shelf life, biochemical attributes, gene expression

## Abstract

Oxidative damage leading to loss of nutritional quality and pericarp discoloration of harvested litchi fruits drastically limits consumer acceptance and marketability. In the present investigation, the impact of postharvest melatonin application at different concentrations, i.e., 0.1 mM, 0.25 mM, and 0.5 mM, on fruit quality and shelf life of litchi fruits under cold storage conditions was studied. The results revealed the positive effect of melatonin application at all concentrations on fruit quality and shelf life. However, treatment with 0.5 mM concentration of melatonin resulted in minimum weight loss, decay loss, pericarp discoloration, and also retained higher levels of TSS, acidity, total sugar, ascorbic acid, anthocyanin, antioxidant, and phenolics content during cold storage. Melatonin administration also restricted the enzymatic activity of the polyphenol oxidase (PPO) and peroxidase (POD) enzymes in the fruit pericarp and maintained freshness of the fruits up to 30 days in cold storage. At the molecular level, a similar reduction in the expression of browning-associated genes, *LcPPO, LcPOD*, and Laccase, was detected in preserved litchi fruits treated with melatonin. Anthocyanin biosynthetic genes, *LcUFGT* and *LcDFR*, on the other hand showed enhanced expression in melatonin treated fruits compared to untreated fruits. Melatonin, owing to its antioxidant properties, when applied to harvested litchi fruits retained taste, nutritional quality and red color pericarp up till 30 days in cold storage.

## 1 Introduction

Litchi (*Litchi chinensis* Sonn.) is a treasured fruit tree of the subtropical region, which belongs to the Sapindaceae family and is endemic to Southern China’s Guangdong and Fujian provinces (Wu et al., 2007). Litchi is recognized as the “queen of subtropical fruit crops” and dominates domestic and international markets during the summer months due to its appealing red pericarp and aromatic, delicious, and nutrient-rich aril (Wang et al., 2020). The fruit is high in phytonutrients such as vitamin C (64 mg/100 g), as well as traces of vitamins A, B, and minerals such as potassium, phosphorus, Ca, Mg, Fe, Zn, and Cu (Nath et al., 2018). A variety of bioactive substances, including flavonoids and anthocyanins, have been identified in pulp and in inedible tissues including seeds, pericarp, blossoms, and leaves. These substances have potent anti-inflammatory and anti-cancer properties due to their ability to scavenge free radicals (Mir and Perveen, 2022). Litchi is a non-climacteric fruit with a short shelf life and restricted availability. Soon after harvesting completely ripe litchi fruits, pericarp browning and desiccation begin, resulting in postharvest losses of 20%–30% on average (Deshi et al., 2020). Thus, the primary postharvest problem that lowers the potential of litchi for both domestic and international markets is pericarp browning. Several potential causes, ranging from 8 to 10 for pericarp browning in litchi, have been proposed, which includes desiccation, anthocyanin degradation, enhanced lipid peroxidation, membrane leakage, loss of antioxidants, overproduction of free radical, and accelerated enzymatic activity of polyphenol oxidase (PPO) and peroxidase (POD) (Zhang et al., 2018). Litchi’s thin pericarp makes it susceptible to water loss, discoloration, and decay, resulting in a reduction in fruit quality and nutritional value (Chen et al., 2019). Since raw consumption of litchi fruit is the norm, research into preservation technologies is critical to enhance postharvest shelf life of litchi fruit by reducing pericarp browning and maintaining fruit quality during storage. Physical methods like controlled atmosphere storage (CAS) (Liu et al., 2011), modified atmosphere packaging (MAP) (Semeerbabu et al., 2007), gamma radiations (Pandey et al., 2013), and chemical treatments like application of 1-methylcyclopropane (De Reuck et al., 2009), sodium metabisulfite (Liang et al., 2012), chitosan 1% (Lin et al., 2011), and nitric oxide (Barman et al., 2014) have been employed in the past to enhance shelf life of litchi fruits and to maintain its freshness. Of all the several techniques employed, only two postharvest methods, SO_2_ fumigation and fungicide dips, have been commercially used to extend the storage life of litchi fruits (Jiang et al., 2003; Mir et al., 2018a). Thus, a more effective and practical method of extending shelf life of litchi fruits is required.

Melatonin, a tryptophan derivative, is an endogenous plant compound having an impact on several plant physiological processes like seed germination, plant growth, flowering, fruit development, ripening, and senescence. It is also well known for its role in the reduction of oxidative damage caused by biotic and abiotic stress (Arnao and Hernández-Ruiz, 2015). Recently, studies conducted to evaluate the impact of melatonin on enhancing shelf life of fruits and vegetables are gaining momentum due to the effectiveness of this safe and novel preservative. In litchi, the major research area remains, extending postharvest shelf life and reducing pericarp browning of the fruit. A study conducted on litchi cv. Baitangying demonstrated that melatonin treatment significantly suppressed pericarp browning of the fruit during refrigerated storage as the chromaticity L*, a*, b* values of the melatonin-treated fruits were 6.2%, 22.4%, and 19.6% higher than those of the control fruits (Liu et al., 2020). Wang et al. (2020) noticed delayed pericarp browning in melatonin-treated fruits compared to untreated fruits in their investigation on litchi cv. A4Wuhe. The compound is thus assumed critical in the shelf life enhancement and preservation of fruit quality of fruits and vegetables by increasing antioxidant capacity, reducing chilling injury, and preventing pathogen growth (Feng et al., 2022). Melatonin postharvest treatment is currently the subject of great interest as it is also effective at quenching free radicals and activating the antioxidant system, thus preventing cumulative oxidative damage to cell membranes. Therefore, the present study was conducted with the aim of ascertaining the impact of different concentrations of melatonin as a postharvest treatment on storage life and nutritional quality of litchi fruits during cold storage. Molecular analysis was also attempted on melatonin-treated fruits, where genes related to pericarp discoloration or browning and anthocyanin synthesis and degradation were targeted, so that the study could also provide an insight into the molecular level about the mechanism of melatonin function. The present study aims to assess the impact of different concentrations of melatonin as a postharvest treatment on the storage life and nutritional quality of litchi fruits during cold storage. The findings are expected to contribute to the development of a new postharvest technique to increase the shelf life of litchi fruits and its availability for a longer period.

## 2 Materials and methods

### 2.1 Plant material and treatments

Litchi cv. Purbi fruits were picked at commercial maturity stage based on color, from the Horticulture Garden of the Department of Fruit & Fruit Technology, Bihar Agricultural College, Sabour, Bihar, in bunches with few leaves to avoid rupturing. The fruits were then taken to the department laboratory for treatment and analysis. The blemished or infected fruits were then discarded from the lot. Fruits of uniform size, shape, and color were selected and washed thoroughly with Tween-20 (0.01%). Later, the fruits were destalked with a sharp blade, to leave a 2-mm-long pedicel. They were then randomly divided into four treatment groups, each consisting of 200 fruits, with five replicates. The first batch of fruits was used as the control and was soaked in distilled water for 10 min. For 10 min each, the second batch of fruits was immersed in 0.1 millimolar (mM) melatonin solution, the third batch was immersed in 0.25 mM melatonin solution, and the fourth batch was immersed in 0.5 mM melatonin solution. After air-drying at room temperature, the fruits were packaged in perforated polyethylene bags. The fruit bags were then stored at 4–7°C and 85%–90% RH. Fruits from each batch were sampled every 3 days. Weight loss, decay loss, and pericarp browning index of litchi fruits were quantified non-destructively, whereas other enzymatic and biochemical analyses were quantified by destructive means.

### 2.2 Determination of weight loss (%), decay loss (%), and pericarp browning index

The weight loss of melatonin-treated and control fruits was calculated by estimating the difference between the initial fruit weight recorded before storing the fruits under cold storage conditions and the final weight of the fruit recorded on every third day of cold storage. This difference in fruit weight was then divided by the initial fruit weight. The percentage of weight loss was then calculated. The decay loss in litchi fruits was visually assessed by inspecting the fruits for evidence of decay caused by micro-organisms on each sampling day. To determine decay loss, the number of fruits exhibiting visible decay sign was divided by the total number of fruits, which was subsequently calculated as percentage (%). The visual evaluation of pericarp browning in litchi fruits was analyzed using the approach of Kumar et al. (2013). The fruits were graded into five categories based on the five-point scale browning score. To calculate the pericarp browning index, the number of fruits in each scale category was multiplied by the corresponding browning score, which was later on divided by the total number of fruits.

### 2.3 Enzymatic assay

The enzyme’s crude extract was made by homogenizing 2 g of litchi pericarp in a 25-mL solution of sodium phosphate buffer (50 mM, pH 7.0) and 0.25 g of polyvinyl pyrrolidone. The resulting supernatant was used for PPO and POD enzymatic analysis. Protocols demonstrated by Jiang (2000) and Jiang and Fu (1999) were followed for estimation of PPO and POD enzyme activity, respectively, in litchi pericarp. For the PPO assay, 2.8 mL of 20 mM catechol solution prepared in sodium phosphate buffer (0.01 M; pH 6.8) was added to 0.2 mL of enzyme extract. For the POD assay, 2.75 mL of phosphate buffer (50 mM; pH 7.0), 0.1 mL of guaiacol (4%), and 0.1 mL of H_2_O_2_ (1%) were added to 0.05 mL of enzyme extract. The absorbance was measured using a UV–Vis spectrophotometer (Dynamica HALO DB-20S, Australia) at 470 nm. A unit of enzymatic activity was defined as the amount of enzyme that produces a 0.1 shift in absorbance minute^−1^ for PPO and 0.01 shift in absorbance minute^−1^ for POD. The enzyme activity was measured in U/g.

### 2.4 Determination of total soluble solids and titratable acidity and total sugar content

The total soluble solid (TSS) of litchi pulp of all the treatments was measured using a digital refractometer at 25°C. At every sampling day, the fruit juice of litchi was extracted by squeezing the pulp with the help of a muslin cloth and was placed on the prism of refractometer and readings were taken. The results were expressed in percentage (%). The titration method was performed to determine the titratable acidity (TA) of the aril (AOAC, 2000) at 3-day intervals. Titration was carried out against a solution of 0.1 N sodium hydroxide, and phenolphthalein dye was used as an indicator. In 10 mL of litchi juice, two to three drops of phenolphthalein dye was added and mixed thoroughly. It was then titrated with 0.1 N NaOH solution from the burette. The appearance of permanent light pink color was considered as the endpoint of titration. The recorded titer value was then used to calculate the TA value as percent (%) of citric acid. The Lane and Eynon (1923) method was used to determine sugar concentrations. Ten grams of crushed fruit sample was hydrolyzed for 24 h at room temperature after being mixed with 5 mL of concentrated HCl. After 24 h, two drops of the phenolphthalein indicator was added, followed by 40% NaOH until a pink color appeared. Then, 0.1 N HCl was added to it drop by drop until the pink color disappeared. Then, distilled water was added to increase the volume to 100 mL. The resulting solution was stored in a burette for titration. Five milliliters of each Fehling’s solutions A and B were combined in a beaker, then the volume was increased to 50 mL by adding distilled water. Two drops of methylene blue was added and the resulting solution was then heated. The titration was performed when the solution began to boil. The appearance of brick red color was considered as the endpoint of titration. The titer values were recorded and the final values were expressed in terms of percentage after calculation.

### 2.5 Determination of ascorbic acid and total anthocyanin content

The ascorbic acid content of the fruit samples was ascertained using the method outlined by Jones and Hughes (1983). A sample extract was prepared by homogenizing 10 g of fruit sample in 3% meta-phosphoric acid solution, then the volume was increased to 100 mL with distilled water and the resulting sample was centrifuged at 10,000 rpm for 20 min. The supernatant obtained was collected and titrated against 2,6-dichlorophenolindophenol dye until the pink endpoint appeared that lasted for 15 s. The ascorbic acid content was calculated using the titer value and expressed in mg/L. For anthocyanin analysis, 1 g of litchi pericarp was added to 9 mL of HCl and methanol solution prepared at a ratio of 15:85. The extraction mixture with the pericarp was kept overnight in a dark room to guarantee complete extraction. Absorbance was measured at 535 nm using a double-beam UV–Vis spectrometer. The anthocyanin content was expressed in mg/kg.

### 2.6 Determination of total phenolic content and antioxidant capacity

Total phenolic content (TPC) of the litchi pericarp was ascertained using the Folin–Ciocalteu reagent (Singleton et al., 1999). The phenols in the sample were extracted in ethanol (80%), and the resultant extract was then centrifuged (Sigma) for 20 min at 10,000 rpm. The acquired supernatant was utilized for the analysis of TPC and antioxidant capacity. A reaction mixture of 0.1 mL of sample extract, 2.9 mL of distilled water, 0.5 mL of Folin–Ciocalteu reagent, and 2.0 mL of Na_2_CO_3_ (20%) was produced and maintained at room temperature for 60 min for TPC detection. The absorbance of the sample was measured at 765 nm using a UV–Vis spectrometer. The results were compared to the gallic acid standard calibration curve and expressed as mg/kg GAE. The antioxidant capacity of the litchi pericarp was estimated using the CUPRAC (cupric reducing antioxidant capacity) assay (Apak et al., 2008). A total volume of 4.1 mL was created by adding 0.1 mL of sample extract and 1 mL of each of the following solutions to a test tube: copper (II) chloride solution, neocuproine alcoholic solution, ammonium acetate buffer solution, and distilled water. Thirty minutes were then spent letting the mixed solution stand. Afterward, in comparison to the reagent blank, the absorbance was measured at 450 nm in a spectrophotometer. The value of antioxidant capacity was given as mmol/kg Trolox of fresh fruit weight.

### 2.7 Molecular analysis: RNA isolation, cDNA synthesis, and qRT-PCR analysis

The total RNA from litchi pericarp of control and postharvest melatonin-treated fruits stored in cold storage facility for 30 days was extracted with slight modifications in the CTAB protocol outlined by Wang et al. (2014). Agar gel electrophoresis and a spectrophotometer were used to analyze the quality and quantity of the extracted RNA, respectively. Single-stranded cDNA (Promega) was synthesized and was used for the PCR study along with actin gene (reference gene) to analyze the relative expression levels of the different genes under study. To determine the effect of postharvest melatonin application on stored litchi fruits (pericarp) at cold storage conditions, two structural genes involved in the anthocyanin biosynthetic pathway of litchi, i.e., *LcDFR* (dihydroflavonol 4-reductase) and *LcUFGT* (UDP-glucose: flavonoid 3-*O*-glucosyltransferase), and anthocyanin degradation, i.e., *Laccase*, were investigated. Conversion of dihydroflavonols to anthocyanin 3-O-glycosides is regulated by *DFR*, *ANS* (anthocyanidin synthase), and *UFGT* genes (Dixon and Palva, 1995). Genes encoding for PPO and POD enzymes, i.e., *LcPPO* (PPO) and *LcPOD* (peroxidase), were also taken into consideration, as these enzymes are responsible for polyphenol oxidation and pericarp browning. Another gene that is responsible for anthocyanin degradation of litchi pericarp is *Laccase*. qRT-PCR (quantitative real-time PCR) was done on a Light Cycler system (Applied Biosystems) using SYBR Dye to examine the transcript levels of the genes under study. Utilizing a primer for each gene, qRT-PCR was quantified in triplicate. **Table 1** lists the primers used in the qRT-PCR analysis. The reaction mixture consisted of diluted cDNA sample as template (2.0 µL), 1 µL each of forward and reverse primers (10 µM), and real-time master mix (5 µL), making a total volume of 20 µL. The PCR conditions are provided in **Supplementary Table 1**.

**Table 1:** Primers for real-time PCR analysis.

Gene	Forward Primer	Reverse Primer
***LcPOD***	GACGACACAT CCACCTTTGT	CCAGGACATGC TTTCTCTAGTT
***LcDFR***	ACGTCATCAGC AGGAACTATG	CAGTCATCTTT ACGGACCTGAC
***LcUFGT***	CTTTCGTGCCT TGTTGGTTATC	GCTGCTCATCT TCTCTTCCTT
***Laccase***	CCAGCCAGTGG AACTACTTATC	CACTGCCTCTT CATAGACTTCC
***Lcactin***	AATGGAACTGG AATGGTCAAG	TGCCAGATCTT CTCCATGTCAT

### 2.8 Statistical analysis

The study was carried out in a completely randomized design (CRD), with four treatments and five replications. Duncan’s multiple range test was used to compare the means of the treatments at a significance level of *p* ≤ 0.05. The molecular study data were statistically analyzed, and the significant differences between the means were calculated by one-way and two-way ANOVA (*p* < 0.001).

## 3 Results and discussion

### 3.1 Weight loss and decay loss

The litchi fruits quickly lose water and nutrients and become susceptible to diseases, and the pericarp color changes from bright red to brown in color. Water loss in litchi has been identified as one of the causes of fruit senescence and atrophy in investigations (Kumari et al., 2015). Reduction in the desiccation rate of the peel can maintain freshness of the fruits during storage. The results pertaining to weight loss exhibit a gradual increase in weight loss during the time span of 30 days under cold storage conditions, although the weight loss percentage was significantly lower in melatonin-dipped fruits than in control fruits (**Table 2**). The reduction in fruit weight was observed from the first day of sampling, i.e., 3 days of storage. Initially, the untreated fruits showed maximum weight loss and the fruits treated with 0.5 mM melatonin exhibited the least weight loss, and the same trend was observed till the last day of cold storage. At the end of the storage period, the control fruits showed maximum weight loss (12.81%) and the least weight loss was recorded in fruits treated with 0.5 mM melatonin (5.77%), which was 2.2-fold less than the control. Weight loss in melatonin-treated fruits was happening at a slower rate compared to control fruits since melatonin treatment is known to preserve cell structure, increase cell rigidity, and boost the process of lignification, all of which substantially reduced respiration (Onik et al., 2021) and transpiration losses. Using 0.2 mmol/L and 0.6 mmol/L of melatonin, Xie et al. (2022) obtained equivalent results of decreased weight loss in “Feizixiao” litchi during storage at 4°C. Reduced weight loss was reported after melatonin treatment in various fruit crops such as peach (Gao et al., 2016), strawberry (Liu et al., 2018), sweet orange (Ma et al., 2021), loquat (Wang et al., 2021), mango (Njie et al., 2022), and rambutan (Wei et al., 2022).

**Table 2:** Postharvest melatonin treatment (MT) effect on weight loss in litchi fruit cv. Purbi during cold storage.

Treatment	Weight loss (%)										
	Storage days										
	0	3	6	9	12	15	18	21	24	27	30
T_1_ (Control)	–	0.57^a^	1.05^a^	2.16^a^	3.28^a^	4.24^a^	5.34^a^	8.39^a^	10.11^a^	11.84^a^	12.81^a^
T_2_ MT (0.1 mM)	–	0.24^a^	0.47^ab^	0.95^b^	1.65^b^	2.12^b^	3.10^b^	4.03^b^	5.79^b^	6.82^b^	7.69^b^
T_3_ MT (0.25 mM)	–	0.15^a^	0.34^b^	0.71^b^	1.25^b^	1.90^b^	2.11^b^	3.69^b^	4.57^c^	5.09^b^	6.51^ab^
T_4_ MT (0.5 mM)	–	0.05^a^	0.12^b^	0.25^b^	0.61^b^	1.21^b^	1.88^b^	2.02^c^	3.00^c^	4.90^b^	5.77^b^

Values in tables indicate mean of five replicates. Different letters in the same column indicate significant differences at p ≤ 0.05 (Duncan’s multiple range test).

The symbol “-“ in **Table 2** means that on the day of harvesting litchi fruits i.e., Day 0, no weight loss was recorded. The fruit weight recorded on Day 0 served as initial fruit weight which was used to calculate weight loss on 3 days interval.

An increased ROS production during fruit storage causes oxidative stress, which causes fruits to decay (Cao et al., 2018) and increased susceptibility to postharvest diseases (Zhang et al., 2021). Early decay incidence was observed in untreated litchi fruits in comparison to the melatonin-treated fruits (**Table 3**). Decay percentage increased with the progression of storage duration; however, melatonin dipping of fruits significantly retarded the decay loss. Melatonin is also a potent antioxidant, which can inhibit reactive oxygen species burst, therefore extending the shelf life of horticulture crops (Ma et al., 2021; Sharafi et al., 2021). Melatonin treatment of fruits delayed decay by 18 days when stored in cold storage. The minimum percent of decay loss (20.00%) after 30 days of cold storage was recorded in 0.5 mM melatonin-treated fruits, which was 4.3-fold lower than the control (86.77%), followed by 0.25 mM (25.00%) and 0.1 mM (28.77%) melatonin-treated fruits that exhibit 3.4-fold and 3.01-fold lower decay loss, respectively, over the untreated fruits. According to Zhang et al. (2021), postharvest treatment with 0.25 mM melatonin for 15 min on litchi fruits reduced the development of litchi downy blight when stored at 25°C, thereby lowering the decay loss. Citrus (Lin et al., 2019), peach (Gao et al., 2016), apple (Yin et al., 2013), and mango (Liu et al., 2020) were other crops where exogenous application of melatonin decreased occurrence of postharvest diseases and decay loss.

**Table 3:** Postharvest melatonin treatment (MT) effect on decay loss in litchi fruit cv. Purbi during cold storage.

Treatment	Decay loss (%)										
	Storage days										
	0	3	6	9	12	15	18	21	24	27	30
T_1_ (Control)	0	0	0	0	16.70^a^	36.70^a^	46.70^a^	63.33^a^	76.77^a^	83.33^a^	86.77^a^
T_2_ MT (0.1 mM)	0	0	0	0	0.0^b^	5.00^b^	11.00^b^	16.77^b^	21.77^b^	24.77^b^	28.77^b^
T_3_ MT (0.25 mM)	0	0	0	0	0.0^b^	0.00^b^	6.77^c^	10.00^c^	16.70^c^	21.33^c^	25.00^c^
T_4_ MT (0.5 mM)	0	0	0	0	0.0^b^	0.00^b^	3.33^d^	6.70^d^	10.33^d^	16.77^d^	20.00^d^

Values in tables indicate mean of five replicates. Different letters in the same column indicate significant differences at p ≤ 0.05 (Duncan’s multiple range test).

### 3.2 Pericarp browning

The first visible sign of fruit deterioration in litchi is pericarp browning, which adversely affects the marketability of the fruits after harvest. An array of biochemical and physiological processes is involved in the process of the litchi pericarp browning. According to Jiang et al. (2004), the pericarp’s phenolic compounds, such as anthocyanins and epicatechin (EC), are oxidized and degraded, which contributes significantly to pericarp browning in litchi. Regardless of treatment, the pericarp browning index in litchi fruits rises with the storage duration (**Table 4**). When compared to other treatments, the control fruits were observed with rapid pericarp browning. Up to the 6th storage day, no browning incidence was observed in any of the treatments. The browning in 0.1 mM melatonin-treated fruits and control fruits started on the 9th day of storage, whereas the fruits treated with 0.25 mM and 0.5 mM melatonin started exhibiting browning symptoms on the 12th day of storage. On the final day of storage (30th day), fruits treated with 0.5 mM melatonin was recorded with minimum browning score (2.50), which was 1.98-fold lower when compared to the score of untreated fruits (4.95). This was followed by 0.25 mM (2.91) and 0.1 mM (3.33) melatonin-treated fruits, which exhibited 1.71-fold and 1.48-fold lower pericarp browning, respectively, when compared to untreated fruits. The results revealed that 0.5 mM melatonin application effectively reduced the extent of pericarp browning along with reduction in the activity of browning enzymes. Various studies involving melatonin treatment has shown to effectively enhance the antioxidant activity, which slows down the oxidation and anthocyanin degradation process, thereby reducing the development of brown spots (Xie et al., 2022). Melatonin therapy has also been shown to successfully inhibit the rate of enzymatic browning in litchi (Zhang et al., 2018; Marak et al., 2023) and rambutan (Wei et al., 2022).

**Table 4:** Postharvest melatonin treatment (MT) effect on pericarp browning index in litchi fruit cv. Purbi during cold storage.

Treatment	Pericarp browning index										
	Storage days										
	0	3	6	9	12	15	18	21	24	27	30
T_1_ (Control)	–	–	–	1.40^a^	1.83^a^	2.82^a^	3.32^a^	4.05^a^	4.58^a^	4.82^a^	4.95^a^
T_2_ MT (0.1 mM)	–	–	–	0.13^b^	0.33^b^	0.93^b^	1.23^b^	1.94^b^	2.33^b^	2.90^b^	3.33^b^
T_3_ MT (0.25 mM)	–	–	–	0.00^b^	0.27^b^	0.57^c^	1.15^b^	1.64^b^	2.09^c^	2.60^c^	3.12^c^
T_4_ MT (0.5 mM)	–	–	–	0.00^c^	0.10^c^	0.27^d^	0.70^c^	1.10^c^	1.63^d^	2.17^d^	2.50^d^

Values in tables indicate mean of five replicates. Different letters in the same column indicate significant differences at p ≤ 0.05 (Duncan’s multiple range test).

### 3.3 Polyphenol oxidase and peroxidase activity

Polyphenol oxidation in the litchi pericarp by enzymes such as PPO and POD begins quickly after harvesting, resulting in dull-brown pericarp (Jiang et al., 2004). As the storage duration progressed, the enzymatic activity of PPO and POD increased in all treatments (**Tables 5**, **6**). Untreated fruits displayed higher enzymatic activity of PPO (346.0 U/g) and POD (299.7 U/g) at the end of the storage period as compared to the melatonin-treated fruits. On the last day of storage, fruits treated with 0.5 mM melatonin exhibited the lowest PPO (234.7 U/g) and POD (171.7 U/g) enzyme activity in the fruit pericarp tissues. PPO and POD have been proven to play an instrumental part in the enzymatic browning of several fruits (Zhang et al., 2018). It oxidizes phenols and produces colored quinine, deepening the browning state. Our investigation clearly indicates lowering of level of PPO and POD enzymes after melatonin treatment and the current study’s findings are substantially comparable to previous reported findings, where exogenous melatonin treatment extended the shelf life of litchi fruits by combating the rapid increase in PPO and POD levels after harvest (Zhang et al., 2018; Marak et al., 2023).

**Table 5:** Postharvest melatonin treatment (MT) effect on PPO activities in litchi fruit cv. Purbi during cold storage.

Treatment	PPO (U/g)										
	Storage days										
	0	3	6	9	12	15	18	21	24	27	30
T_1_ (Control)	100	107.3^a^	125.0^a^	139.0^a^	145.0^a^	185.0^a^	225.0^a^	255.0^a^	290.0^a^	327.0^a^	346.0^a^
T_2_ MT (0.1 mM)	100	104.0^b^	115.7^b^	126.7^b^	142.7^b^	167.7^b^	189.7^b^	237.7^b^	271.7^b^	288.7^b^	296.7^b^
T_3_ MT (0.25 mM)	100	104.7^b^	114.7^b^	124.5^b^	140.7^c^	165.7^b^	185.7^b^	225.7^c^	259.7^c^	273.7^c^	293.7^c^
T_4_ MT (0.5 mM)	100	101.3^c^	107.7^c^	116.7^c^	130.7^d^	149.7^c^	171.7^c^	194.7^d^	207.7^d^	221.7^d^	234.7^d^

Values in tables indicate mean of five replicates. Different letters in the same column indicate significant differences at p ≤ 0.05 (Duncan’s multiple range test).

**Table 6:** Postharvest melatonin treatment (MT) effect on POD activities in litchi fruit cv. Purbi during cold storage.

Treatment	POD (U/g)										
	Storage days										
	0	3	6	9	12	15	18	21	24	27	30
T_1_ (Control)	66	68.7^a^	75.6^a^	107.7^a^	130.7^a^	163.7^a^	195.1^a^	225.7^a^	245.7^a^	270.7^a^	299.7^a^
T_2_ MT (0.1 mM)	66	66.30^b^	68.20^b^	72.10^b^	93.3^b^	106.3^b^	120.3^b^	145.3^b^	163.3^b^	197.3^b^	223.3^b^
T_3_ MT (0.25 mM)	66	66.30^b^	68.30^b^	72.30^b^	90.3^b^	104.3^bc^	115.3^c^	130.3^c^	151.3^c^	176.3^c^	201.3^c^
T_4_ MT (0.5 mM)	66	66.70^b^	68.20^b^	71.70^b^	84.70^c^	96.74^c^	107.7^d^	119.7^d^	136.7^d^	151.7^d^	171.7^d^

Values in tables indicate mean of five replicates. Different letters in the same column indicate significant differences at p ≤ 0.05 (Duncan’s multiple range test).

### 3.4 TSS, titratable acidity, and total sugar

TSS, acidity, ascorbic acid, total sugar, anthocyanin, phenolics, and antioxidants are some of the fruit quality components in litchi that degrade quickly as soon as the fruits are harvested. The eating quality of litchi fruits is mainly governed by TSS, TA, and sugars present. The control and melatonin-treated fruits differed greatly in TSS, TA, and total sugar content at the end of the cold storage period. TSS mostly consists of sugars, as well as various salts, pigments, and organic acids. It is the most crucial element in determining fruit quality. In our study, we discovered that the TSS of litchi fruits increased up to the sixth day of cold storage in all treatments before rapidly declining (**Table 7**). At the end of the storage period, the fruits treated with 0.5 mM melatonin (17.2%) were recorded with the highest TSS value, followed by the fruits treated with 0.25 mM (16.3%) and 0.1 mM (15.6%). The control fruits had the lowest TSS value (13.1%). Throughout the 30-day cold storage period, it was observed that the TA values of all the treatments were steadily declining (**Table 8**). At the conclusion of storage period, the melatonin-treated fruits displayed higher TA levels than the control fruits (0.19%) at all concentrations. Fruits treated with 0.5 mM melatonin had maximum TA on the last day of storage (0.44%), which was 2.32 times greater than the control. Up until 15 days of cold storage, the total sugar content of the stored litchi fruits increased; however, from the 15th day to the 30th day of storage, the total sugar content gradually decreased (**Table 9**). The highest total sugar content (11.10%) was found in fruits treated with melatonin at a concentration of 0.5 mM, which was 1.54 times higher than the control. Fruits treated with 0.25 mM melatonin had a total sugar content of 10.20%, which was statistically at par with 0.1 mM melatonin-treated fruits (10.01%) on the last day of storage. Fruits used as controls had the lowest total sugar content (7.19%). The outcome of our investigation shows that melatonin administration has a positive impact on the TSS, TA, and total sugar content of preserved fruits and treatment with 0.5 mM melatonin retained the highest content of TSS, TA, and total sugar. Several other researchers have also reported that melatonin administration delayed the decline of TSS during storage (Miranda et al., 2020; Nasser et al., 2022). Similarly, administration of melatonin in strawberries (Liu et al., 2018), pitaya (Ba et al., 2021), kiwi fruit (Wang et al., 2019), jujube (Wang et al., 2022), and citrus navel orange (Ma et al., 2021) during postharvest storage resulted in less degradation of TSS and TA. This slow decline in TSS and TA might be attributed to melatonin’s ability to reduce respiration and senescence, thus preserving soluble solids, carbohydrates, and organic acids.

**Table 7:** Postharvest melatonin treatment (MT) effect on TSS in litchi fruit cv. Purbi during cold storage.

Treatment	TSS (%)										
	Storage days										
	0	3	6	9	12	15	18	21	24	27	30
T_1_ (Control)	20.0	20.4^a^	20.5^b^	20.0^b^	19.4^c^	18.2^b^	17.0^c^	16.2^c^	15.3^c^	14.7^d^	13.1^d^
T_2_ MT (0.1 mM)	20.0	20.7^a^	20.8^a^	20.2^b^	20.0^b^	19.2^ab^	18.3^b^	17.7^b^	17.1^b^	15.9^c^	15.6^c^
T_3_ MT (0.25 mM)	20.0	20.6^a^	20.9^a^	20.3^b^	20.2^ab^	19.7^a^	19.2^a^	17.9^b^	17.3^b^	16.8^b^	16.3^b^
T_4_ MT (0.5 mM)	20.0	20.5^a^	20.9^a^	20.6^a^	20.4^a^	20.2^a^	19.5^a^	18.9^a^	18.2^a^	17.7^a^	17.2^a^

Values in tables indicate mean of five replicates. Different letters in the same column indicate significant differences at p ≤ 0.05 (Duncan’s multiple range test).

**Table 8:** Postharvest melatonin treatment (MT) effect on titratable acidity in litchi fruit cv. Purbi during cold storage.

Treatment	Titratable acidity (%)										
	Storage days										
	0	3	6	9	12	15	18	21	24	27	30
T_1_ (Control)	0.7	0.68^a^	0.64^b^	0.58^b^	0.51^b^	0.47^b^	0.41^b^	0.38^b^	0.31^c^	0.25^c^	0.19^c^
T_2_ MT (0.1 mM)	0.7	0.69^a^	0.65^ab^	0.61^ab^	0.55^ab^	0.51^ab^	0.46^ab^	0.41^ab^	0.37^b^	0.31^b^	0.26^b^
T_3_ MT (0.25 mM)	0.7	0.69^a^	0.69^ab^	0.67^ab^	0.62^ab^	0.57^ab^	0.51^ab^	0.46^ab^	0.40^b^	0.34^b^	0.27^b^
T_4_ MT (0.5 mM)	0.7	0.70^a^	0.69^a^	0.68^a^	0.65^a^	0.62^a^	0.58^a^	0.56^a^	0.53^a^	0.48^a^	0.44^a^

Values in tables indicate mean of five replicates. Different letters in the same column indicate significant differences at p ≤ 0.05 (Duncan’s multiple range test).

**Table 9:** Postharvest melatonin treatment (MT) effect on total sugar content in litchi fruit cv. Purbi during cold storage.

Treatment	Total sugar content (%)										
	Storage days										
	0	3	6	9	12	15	18	21	24	27	30
T_1_ (Control)	11.0	11.50^a^	11.8^b^	12.55^b^	13.75^c^	14.10^b^	12.15^c^	13.21^c^	11.20^c^	9.10^c^	7.19^c^
T_2_ MT (0.1 mM)	11.0	11.60^a^	12.10^ab^	13.50^ab^	15.10^b^	16.35^ab^	15.90^b^	13.92^b^	12.01^b^	10.51^b^	10.01^b^
T_3_ MT (0.25 mM)	11.0	11.60^a^	12.20^ab^	13.70^ab^	15.20^b^	16.50^ab^	16.00^b^	14.00^b^	12.60^a^	10.75^ab^	10.20^b^
T_4_ MT (0.5 mM)	11.0	11.70^a^	12.50^a^	14.40^a^	16.30^a^	17.10^a^	16.35^a^	14.52^a^	12.70^a^	11.40^a^	11.10^a^

Values in tables indicate mean of five replicates. Different letters in the same column indicate significant differences at p ≤ 0.05 (Duncan’s multiple range test).

### 3.5 Ascorbic acid

The two main antioxidant systems in litchi fruit are ascorbic acid found in the pulp and polyphenols found in the pericarp. Regardless of treatment, the ascorbic acid concentration in litchi fruits declined throughout the period of 30 days of cold storage (**Table 10**). Melatonin-treated fruits had a higher ascorbic acid concentration at the end of the storage period than control fruits. Melatonin 0.5 mM-treated fruits preserved the highest ascorbic acid concentration on the last storage day (26.60 mg/L), which was 1.85 times higher than the control (14.37 mg/L). This was followed by 0.25 mM (23.87 mg/L) and 0.1 mM (22.10 mg/L) melatonin-treated fruits. Melatonin treatment has been demonstrated to boost the fruit’s tolerance to oxidative stress during ripening and storage by enhancing bioactive substances like ascorbic acid. Comparable to our study, Bal (2021) reported that melatonin doses maintained a higher ascorbic acid content in nectarines, blueberries (Shang et al., 2021), and peaches (Gao et al., 2016; Cao et al., 2018).

**Table 10:** Postharvest melatonin treatment (MT) effect on ascorbic acid in litchi fruit cv. Purbi during cold storage.

Treatment	Ascorbic acid (mg/L)										
	Storage days										
	0	3	6	9	12	15	18	21	24	27	30
T_1_ (Control)	340.0	330.7^b^	325.7^b^	320.7 ^b^	314.7^b^	303.7^c^	282.7^c^	251.7^c^	213.7^c^	173.7^d^	143.7^d^
T_2_ MT (0.1 mM)	340.0	339.7^a^	339.5^a^	337.4^ab^	331.0^ab^	323.0^b^	322.5^b^	302.0^b^	281.0^b^	251.5^c^	221.0^c^
T_3_ MT (0.25 mM)	340.0	339.6^a^	339.4^a^	337.7^ab^	330.6^ab^	323.7^b^	323.1^b^	303.7^b^	282.7^b^	263.2^b^	238.7^b^
T_4_ MT (0.5 mM)	340.0	340.0^a^	339.8^a^	339.0^a^	337.0^a^	332.0^a^	327.0^a^	315.5^a^	296.0^a^	284.0^a^	266.0^a^

Values in tables indicate mean of five replicates. Different letters in the same column indicate significant differences at p ≤ 0.05 (Duncan’s multiple range test).

### 3.6 Total phenolic content and antioxidant capacity

Melatonin is an excellent antioxidant that reduces the level of free radicals in the plant tissues (Arnao and Hernández-Ruiz, 2015) and boosts the preservation of other non-enzymatic antioxidant compounds such as anthocyanin, phenols, and flavonoids (Xie et al., 2022). The TPC decreased over time, regardless of melatonin treatment (**Table 11**). TPC value, on the other hand, decreased faster in control fruits than in melatonin-treated fruits. At the end of the storage period, fruits treated with 0.5 mM melatonin had the highest TPC value (650 mg/kg), followed by fruits treated with 0.25 mM (636 mg/kg) and 0.1 mM (550 mg/kg). Melatonin treatment had a substantial impact on the amount of antioxidant (AOX) capacity in litchi fruits during cold storage (**Table 12**). The AOX capacity of fruits treated with melatonin dropped at a slower rate than the control fruits at all doses. On the final day of observation, fruits treated with 0.5 mM melatonin had the highest AOX capacity (15.23 mmol/kg), which was statistically at par to fruits treated with 0.25 mM melatonin (14.70 mmol/kg) followed by 0.1 mM melatonin treatment (14.30 mmol/kg). In the current investigation, melatonin treatment reduced the decline of TPC, AOX, and total anthocyanin content in litchi fruits as compared to the control. Exogenous melatonin has been shown to enhance the postharvest fruit antioxidant system, primarily by triggering endogenous melatonin production, raising antioxidant enzyme activity, and encouraging the two-way recycling of some antioxidants (Sharafi et al., 2021). Zhang et al. (2018) study revealed that melatonin treatment increased the antioxidant potential of litchi fruits with greater phenolic compounds. Similarly, Liu et al. (2018) also reported that strawberry postharvest life was prolonged due to enhanced phenolic and flavonoid content when treated with melatonin.

**Table 11:** Postharvest melatonin treatment (MT) effect on total phenolic content in litchi fruit cv. Purbi during cold storage.

Treatment	Total phenolic content (mg/kg GAE)										
	Storage days										
	0	3	6	9	12	15	18	21	24	27	30
T_1_ (Control)	8.70	8.53^b^	8.11^b^	7.79^c^	7.39^c^	6.99^d^	6.84^d^	6.30^d^	5.89^d^	5.39^d^	5.19^d^
T_2_ MT (0.1 mM)	8.70	8.69^a^	8.57^a^	8.20^b^	7.70^c^	7.50^c^	7.20^c^	6.70^c^	6.50^c^	5.72^c^	5.50^c^
T_3_ MT (0.25 mM)	8.70	8.67^a^	8.56^a^	8.21^b^	7.81^b^	7.66^b^	7.43^b^	6.96^b^	6.76^b^	6.42^b^	6.36^b^
T_4_ MT (0.5 mM)	8.70	8.69^a^	8.60^a^	8.40^a^	8.10^a^	7.80^a^	7.60^a^	7.30^a^	7.00^a^	6.60^a^	6.50^a^

Values in tables indicate mean of five replicates. Different letters in the same column indicate significant differences at p ≤ 0.05 (Duncan’s multiple range test).

**Table 12:** Postharvest melatonin treatment (MT) effect on antioxidant capacity in litchi fruit cv. Purbi during cold storage.

Treatment	Antioxidant capacity (mmol Trolox equivalent/kg)										
	Storage days										
	0	3	6	9	12	15	18	21	24	27	30
T_1_ (Control)	18.00	17.85^a^	16.90^b^	16.20^ab^	15.30^b^	14.83^b^	14.37^b^	13.87^b^	13.27^b^	12.77^c^	12.20^c^
T_2_ MT (0.1 mM)	18.00	17.97^a^	17.84^a^	16.70^ab^	16.10^a^	15.80^ab^	15.40^ab^	14.90^ab^	14.70^ab^	14.60^ab^	14.30^b^
T_3_ MT (0.25 mM)	18.00	17.93^a^	17.83^a^	16.83^ab^	16.23^a^	16.13^a^	15.73^ab^	15.23^a^	15.03^a^	14.93^a^	14.70^a^
T_4_ MT (0.5 mM)	18.00	17.97^a^	17.85^a^	17.33^a^	16.73^a^	16.53^a^	16.13^a^	15.62^a^	15.42^a^	15.3^a^	15.23^a^

Values in tables indicate mean of five replicates. Different letters in the same column indicate significant differences at p ≤ 0.05 (Duncan’s multiple range test).

### 3.7 Anthocyanin content

One of the key components related with the development of browning has been proven to be the degradation of anthocyanins (Jiang, 2000; Zhang et al., 2005). The steady drop in anthocyanin content caused a decrease in antioxidant ability, thereby hastening the browning of the litchi fruit pericarp. During postharvest storage, anthocyanin content gradually decreased regardless of the melatonin treatments, but the decrease in melatonin-treated fruits was much slower than the untreated ones (**Table 13**). Fruits treated with 0.5 mM melatonin had the highest anthocyanin content (32.63 mg/kg), which was 2.22 times greater than the control (14.70 mg/kg), followed by fruits treated with 0.25 mM (29.60 mg/kg) and 0.1 mM (25.39 mg/kg) on the last day of the experiment. In order to keep the anthocyanin content in the pericarp of litchi fruit for a longer period of time and avoid pericarp discoloration during cold storage, melatonin treatment was found to be very efficient. The bright peel color of litchi is attributed to the anthocyanin pigment (Zhang et al., 2018). Comparable to our findings, Xie et al. (2022) reported that 0.2 and 0.6 mmol/L MT treatment in “Feizixiao” litchi preserved anthocyanin content at 4°C storage. Likewise, in other crops like strawberries (Liu et al., 2018), pomegranates (Jannatizadeh et al., 2019), blueberry (Shang et al., 2021), and litchi (Marak et al., 2023), melatonin treatment retained anthocyanin pigment.

**Table 13:** Postharvest melatonin treatment (MT) effect on total anthocyanin content in litchi fruit cv. Purbi during cold storage.

Treatment	Total anthocyanin content (mg/kg)										
	Storage days										
	0	3	6	9	12	15	18	21	24	27	30
T_1_ (Control)	4.00	3.92^b^	3.83^c^	3.65^b^	3.53^b^	3.27^c^	2.90^c^	2.54^d^	2.13^d^	1.77^d^	1.47^d^
T_2_ MT (0.1 mM)	4.00	3.98^ab^	3.94^c^	3.84^ab^	3.66^ab^	3.54^b^	3.31^b^	3.14^c^	2.94^c^	2.72^c^	2.54^c^
T_3_ MT (0.25 mM)	4.00	4.00^a^	3.97^a^	3.91^a^	3.84^a^	3.78^a^	3.64^a^	3.52^b^	3.36^b^	3.18^b^	2.96^b^
T_4_ MT (0.5 mM)	4.00	4.00^a^	3.98^a^	3.93^a^	3.88^a^	3.82^a^	3.71^a^	3.66^a^	3.53^a^	3.42^a^	3.26^a^

Values in tables indicate mean of five replicates. Different letters in the same column indicate significant differences at p ≤ 0.05 (Duncan’s multiple range test).

### 3.8 Molecular insights into the role of postharvest melatonin treatment on pericarp browning

**Figure 1:**
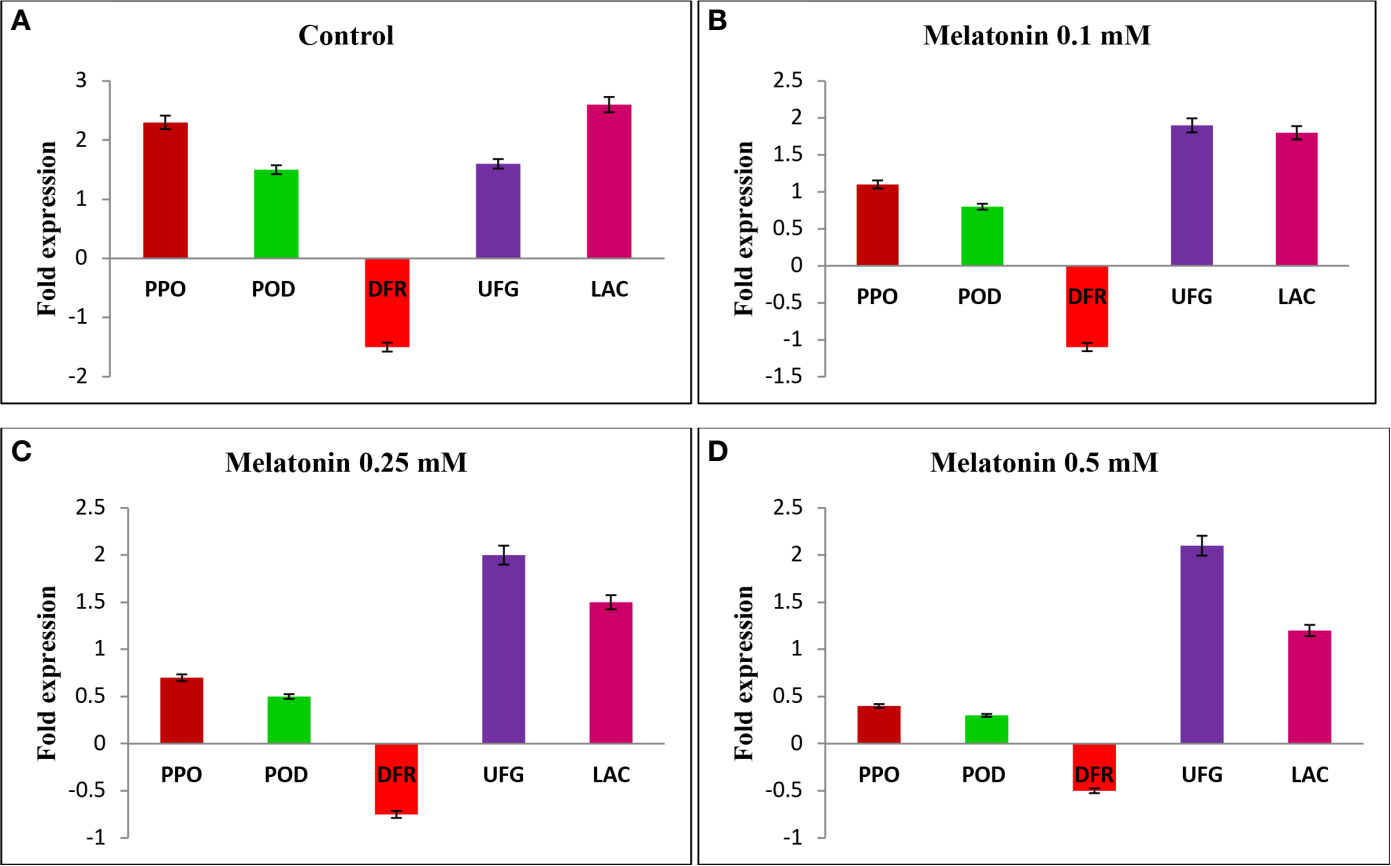
The expression of genes involved in pericarp browning of litchi fruits. **(A)** Control, **(B)** Melatonin at 0.1 mM, **(C)** Melatonin at 0.25 mM, **(D)** Melatonin at 0.5 mM on the 30th day of postharvest cold storage. *Lcactin* gene was used as reference gene during qRT-PCR analysis.

Biotechnology holds great promise in litchi shelf life improvement where the use of these tools and techniques is still in infancy (Mir et al., 2018b). At the end of a 30-day cold storage period, we investigated the relative expression of genes associated with pericarp browning, including *LcPPO, LcPOD*, and *Laccase*, as well as anthocyanin synthesis genes like *LcDFR* and *LcUFGT*, in the pericarp tissue of fruits treated with melatonin at various concentrations and control fruits. The results showed that the levels of *LcPPO, LcPOD*, and *Laccase* gene transcripts were upregulated in all the treatments, with the greatest fold increase in control samples (**Figure 1A**), followed by 0.1 mM melatonin-treated fruits (**Figure 1B**). However, the lowest level of expression of *LcPPO*, *LcPOD*, and *Laccase* gene was exhibited by fruits treated with 0.5 mM melatonin (**Figure 1D**). Compared to the control, melatonin-treated fruits showed an increased level of transcript for the anthocyanin biosynthesis-related genes *LcDFR* and *LcUFGT*. The expression level of *LcDFR* and *LcUFGT* genes was the highest in 0.5 mM melatonin-treated fruits followed by 0.25 mM melatonin-treated fruits, and it was the lowest in the untreated fruits. The upregulation of *LcPPO* and *LcPOD* genes during the postharvest storage of litchi fruits could be attributed to enhanced PPO and POD activity, resulting in the oxidation of polyphenols in the pericarp tissue, which ultimately amplifies pericarp browning (**Figure 1**). In the present study, an enhanced expression of anthocyanin biosynthesis genes, i.e., *LcDFR* and *LcUFGT*, and a reduced expression of an anthocyanin degradation gene, i.e., *Laccase*, were observed in melatonin-treated fruits compared to the control fruits. The increased expression of anthocyanin biosynthesis genes and the reduced expression of the anthocyanin degrading gene after melatonin treatment can be associated with slower degradation of anthocyanin content after melatonin treatment. However, a significant reduction in expression of genes encoding for oxidative enzymes, i.e., *LcPPO* and *LcPOD*, was observed after melatonin treatment was due to less enzymatic browning. A similar result with respect to expression of *LcPPO, LcPOD, LcDFR*, and *LcUFGT* genes was obtained in one of our conducted studies on melatonin-treated litchi fruits when stored at ambient conditions (Marak et al., 2023). Zhang et al. (2018) investigated the molecular mechanism behind delayed pericarp browning of litchi fruits when treated with melatonin and observed that melatonin inhibited the activity of enzymes such as PPO and POD, which were identified as the principal browning agent. This was attributable to the fact that melatonin treatment increased the expression of genes involved in the repair of oxidized proteins, such as *LcMsrA1, LcMsrA2, LcMsrB1*, and *LcMsrB2*. Another possible explanation for delayed senescence and less browning is an increase in the fruit’s internal melatonin content as a result of exogenous melatonin treatment (Liu et al., 2018; Zhang et al., 2018), which would contribute to the fruit’s antioxidant capacity.

Melatonin’s capacity to boost the plant’s antioxidant system as well as its activity as a powerful ROS scavenger has promoted this molecule as an alternative to existing preservative agents used for postharvest storage of fresh fruit. Because litchi fruits are non-climacteric in nature, the process of fruit deterioration starts soon after the harvest, causing loss of the attractive red color, flavor, and nutrients, thus limiting the postharvest shelf life due to pericarp browning and loss of nutritional quality. The results of the present investigation demonstrated that the shelf life of litchi could be extended up to 30 days under cold storage conditions (4–7°C) with postharvest application of melatonin. Among all the concentrations of melatonin used, the findings suggest that the application of 0.5 mM melatonin could be a promising preservation strategy for the litchi fruits. The treatment successfully preserved litchi freshness and its physicochemical qualities compared to untreated fruits with lesser pericarp browning owing to its role as an antioxidant. The expression analysis of browning and anthocyanin degradation-related genes such as *LcPPO*, *LcPOD*, and *Laccase* in control and melatonin-treated fruits made it clear that melatonin treatment of litchi fruits led to reduced expression browning and anthocyanin degradation-related genes. Our approach revealed that postharvest application of melatonin could be a tool for overcoming the problem of pericarp browning in harvested litchi fruits along with extending its availability in the form of stored fruits. Future studies can be directed towards deciphering the genetic mechanism and hormonal crosstalk after postharvest melatonin application.

## Data availability statement

The original contributions presented in the study are included in the article/**Supplementary Material**, further inquiries can be directed to the corresponding authors.

## Author contributions

KM: Data curation, Investigation, Formal analysis, Writing – original draft. HM: Conceptualization, Methodology, Investigation, Supervision, Validation, Project administration, Writing – original draft, Writing – review & editing. MS: Conceptualization, Methodology, Supervision, Writing – review & editing. PS: Methodology, Supervision, Validation, Writing – original draft. FH: Formal analysis, Software, Writing – review & editing. SA: Formal analysis, Funding acquisition, Project administration, Validation, Writing – review & editing.
