# Supplementary material for: Exogenous melatonin delays oxidative browning in litchi during cold storage by regulating biochemical attributes and gene expression
Source: Front Plant Sci. 2024 Jun 6;15:1402607. doi: 10.3389/fpls.2024.1402607 (PMC11187992; doi:10.3389/fpls.2024.1402607)
Supplement: Supplementary file 1 [file Table_1.docx]

**Supplementary Table 1.qRT-PCR reaction conditions**

| **Sl. No.** | **Steps** | **Primers** | | **Cycles** |
| --- | --- | --- | --- | --- |
|  |  | **Temperature (°C)** | **Time** |  |
| 1 | Initial denaturation | 94 | 3 min | 35 cycles |
| 2 | Denaturation | 94 | 30 sec |  |
| 3 | Annealing | 60 for internal control Actin; 52 for primers, *LcPPO,LcPOD, Laccase, LcDFR*and*LcUFGT* | 30 sec |  |
| 4 | Primer extension | 72 | 1min |  |
| 5 | Melt curve | Set to default |  |  |
